# Morphological description of the alimentary tract of *Geoicautricularia* (Passerini, 1856) (Insecta, Hemiptera, Eriosomatinae)

**DOI:** 10.1007/s00435-016-0313-z

**Published:** 2016-05-09

**Authors:** E. Mróz, D. Kertowska, A. Nowińska, B. Baran, P. Węgierek, Ł. Depa

**Affiliations:** Department of Zoology, Faculty of Biology and Environmental Protection, University of Silesia, Bankowa 9, 40-007 Katowice, Poland

**Keywords:** Histology, Aphid, Digestive system, Feeding, Mutualism

## Abstract

Existing literature data report the lack of stomach and crenated intestine in the aphid species *Geoicasetulosa* (Passerini, 1860), a representative of subfamily Eriosomatinae. This odd anatomical feature seemed remarkable, due to the presence of fully developed intestine in closely related genera and mutualistic relationship with ants of this genus. The study aimed at repeated anatomical research of *Geoicautricularia* (Passerini 1856), in order to confirm what seemed to be a generic feature. Standard histological methods were applied, with addition of oblique light microscopy, fluorescence microscopy and confocal laser scanning microscopy. The results indicated the existence of a fully developed intestine, with broad sac-shaped stomach and loops of the crenated intestine. The general anatomy of the alimentary tract of *G*. *utricularia* resembles that of other representatives of the tribe *Fordini*. Also well-developed rectal gland is present, most probably playing a role in modifying the carbohydrate composition of excreted honeydew.

## Introduction

In all aphid species, the ectodermal part of the anterior region of the alimentary tract consists of the stylet bundle, pharynx, foregut and oesophageal valve. The pharynx consists of the pharyngeal duct, valve and pump (Ponsen 2006). The latter works as a sucking pump, which enables feeding on phloem sap (Ponsen 1987). The midgut is the endodermal part of the alimentary tract consisting of the stomach, crenated intestine and descending intestine. The midgut is responsible for production and excretion of enzymes and the absorption of nutritional substances. In this part of the alimentary tract, often the so-called filter chamber develops (Ponsen 1987). It is an adaptation to feeding on the phloem sap, and it takes part in regulation of the osmotic pressure due to the high concentration of sugars in the sap (Rhodes et al. 1997). In various groups of aphids, where the filter chamber is present, it is developed variously, e.g. in the subfamily Lachninae, the proximal part of the midgut is coiled inside the chamber, creating loops (Klimaszewski and Wojciechowski 1976, 1979; Ponsen 1991). Despite the high diversity of the structure of the filter chamber, it is known that foregut and hindgut are connected, so showing the continuity of the tract. In species without the filter chamber, the coiled segments of hindgut adhere the stomach, e.g. *Acyrthosiphonpisum* (Harris 1776) (Dixon 1998; Shakesby et al. 2009). The wall of the descending intestine is internally made up of epithelium, whose cells are adapted to resorption of water, amino-acids and minerals and ends with rectum.

In representatives of Eriosomatinae, the foregut is a simple and straight canal of various lengths, passing into the stomach, which is placed centrally within the body, from 2nd or 3rd thoracic segment (Ponsen 1991). There is no filter chamber. The very short ectodermal part of the posterior region of the alimentary tract consists of a rectum, epidermal invagination and an anal opening.

**Fig. 1:**
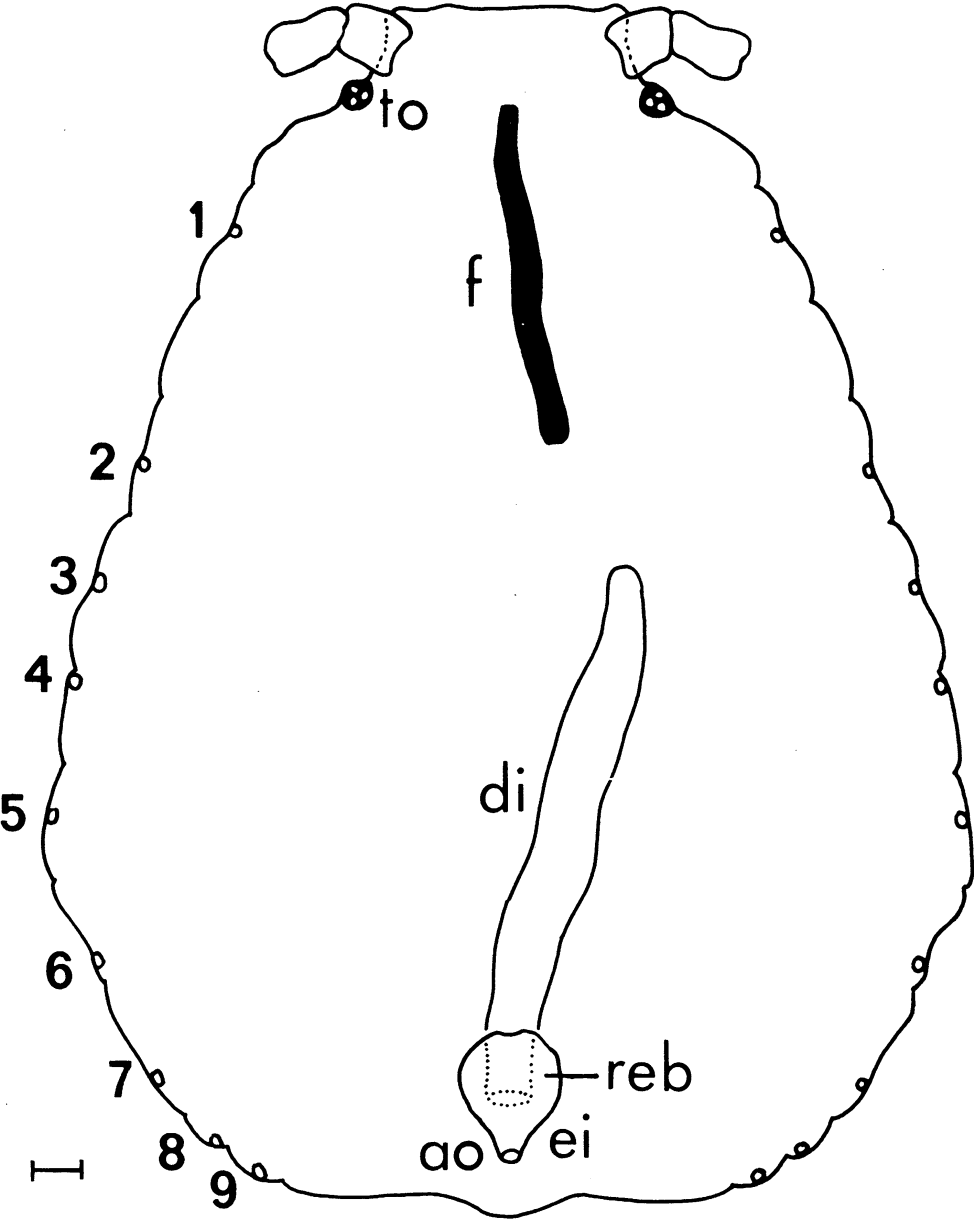
Scheme of alimentary tract of *Geoica* *setulosa* according to Ponsen (1991); *to* triommatidion, *f* foregut, *di* descending intestine, *reb* rectal bladder, *ei* epidermal invagination, *ao* anal opening

**Fig. 2:**
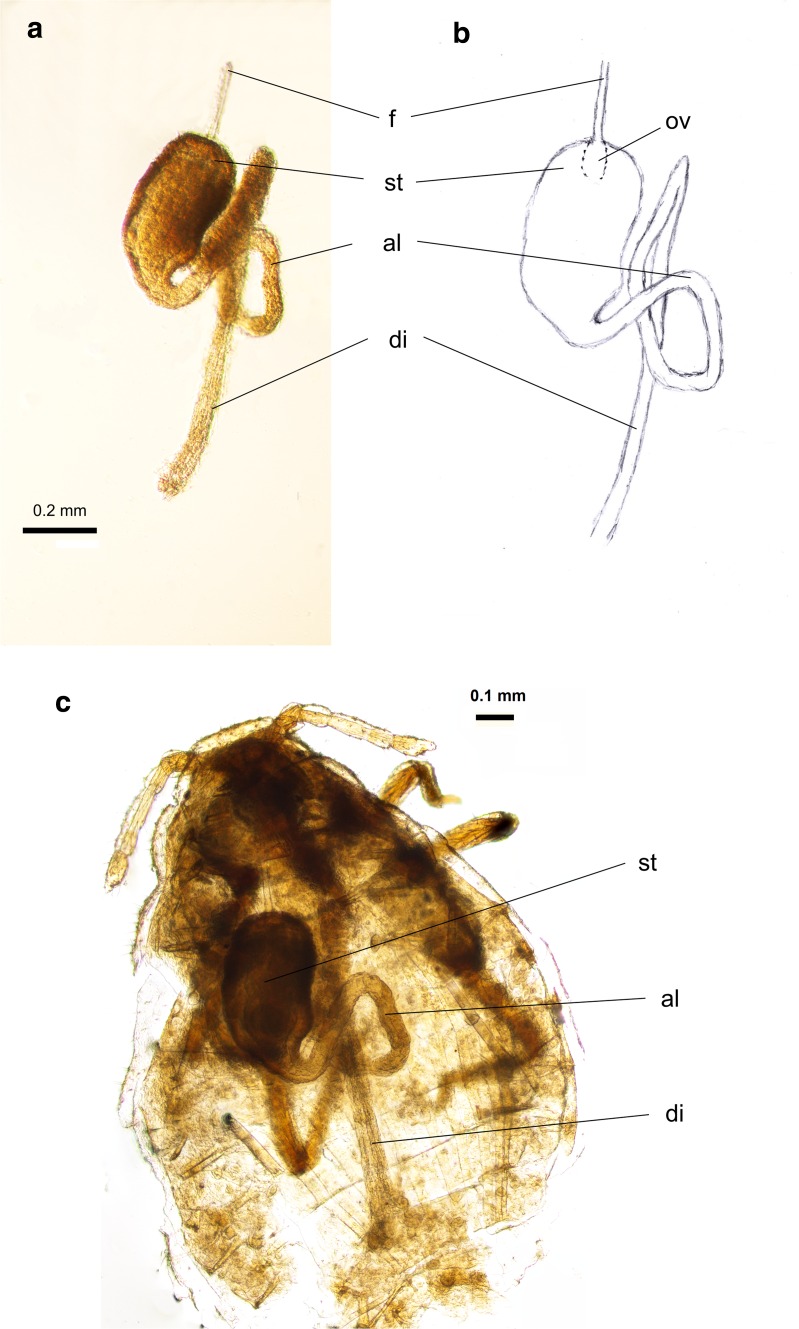
Alimentary tract of *G*. *utricularia*: **a** extracted from the body; **b** schematic reconstruction; **c** location within the body; *f* foregut, *ov* oesophageal valve, *st* stomach, *al* abdominal loop, *di* descending intestine

**Fig. 3:**
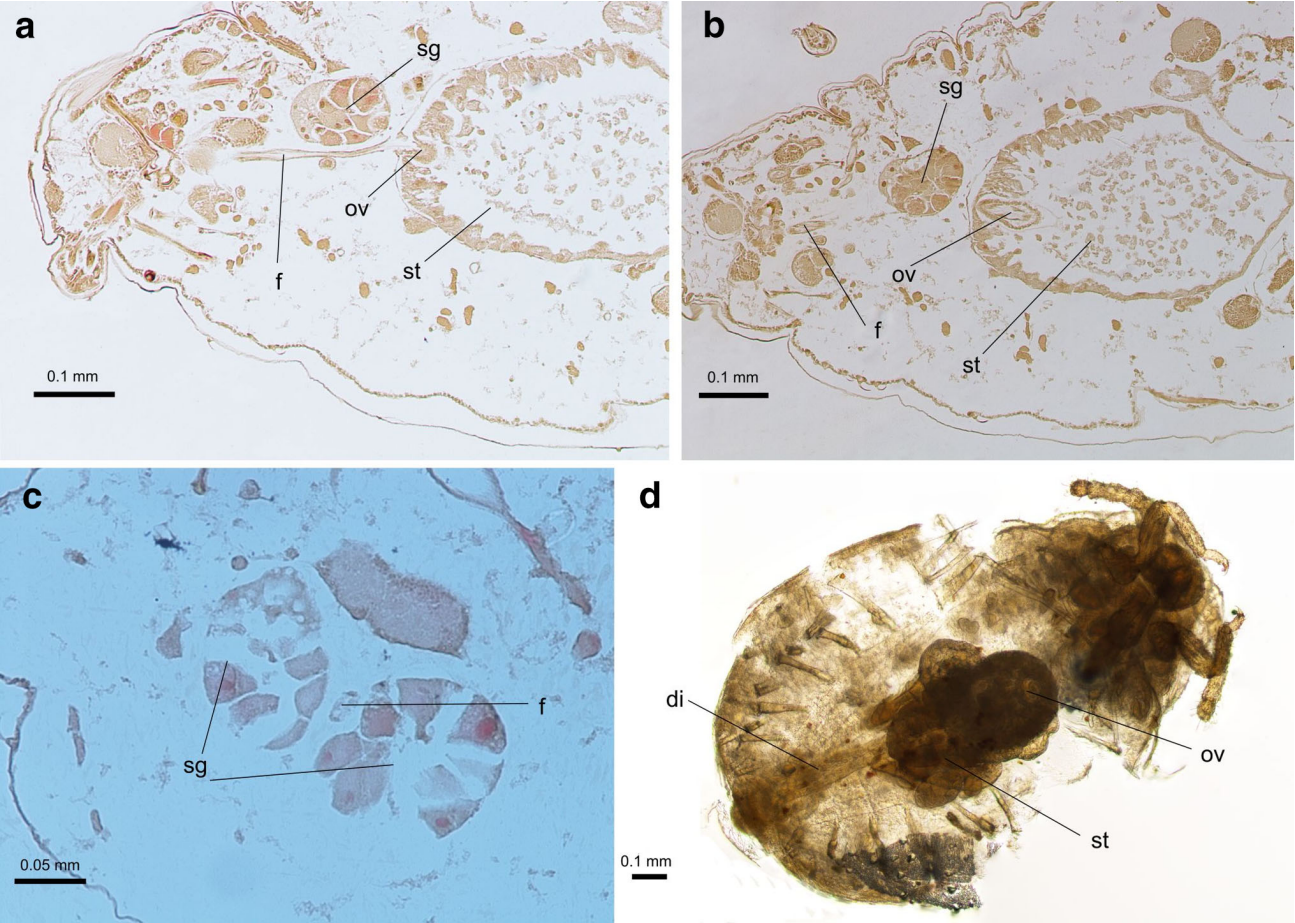
Alimentary tract of *G*. *utricularia*: **a**, **b** longitudinal section through head and thorax; **c** transverse section through salivary glands; **d** location of stomach within the body; *f* foregut, *sg* salivary glands, *ov* oesophageal valve, *st* stomach, *di* descending intestine

**Fig. 4:**
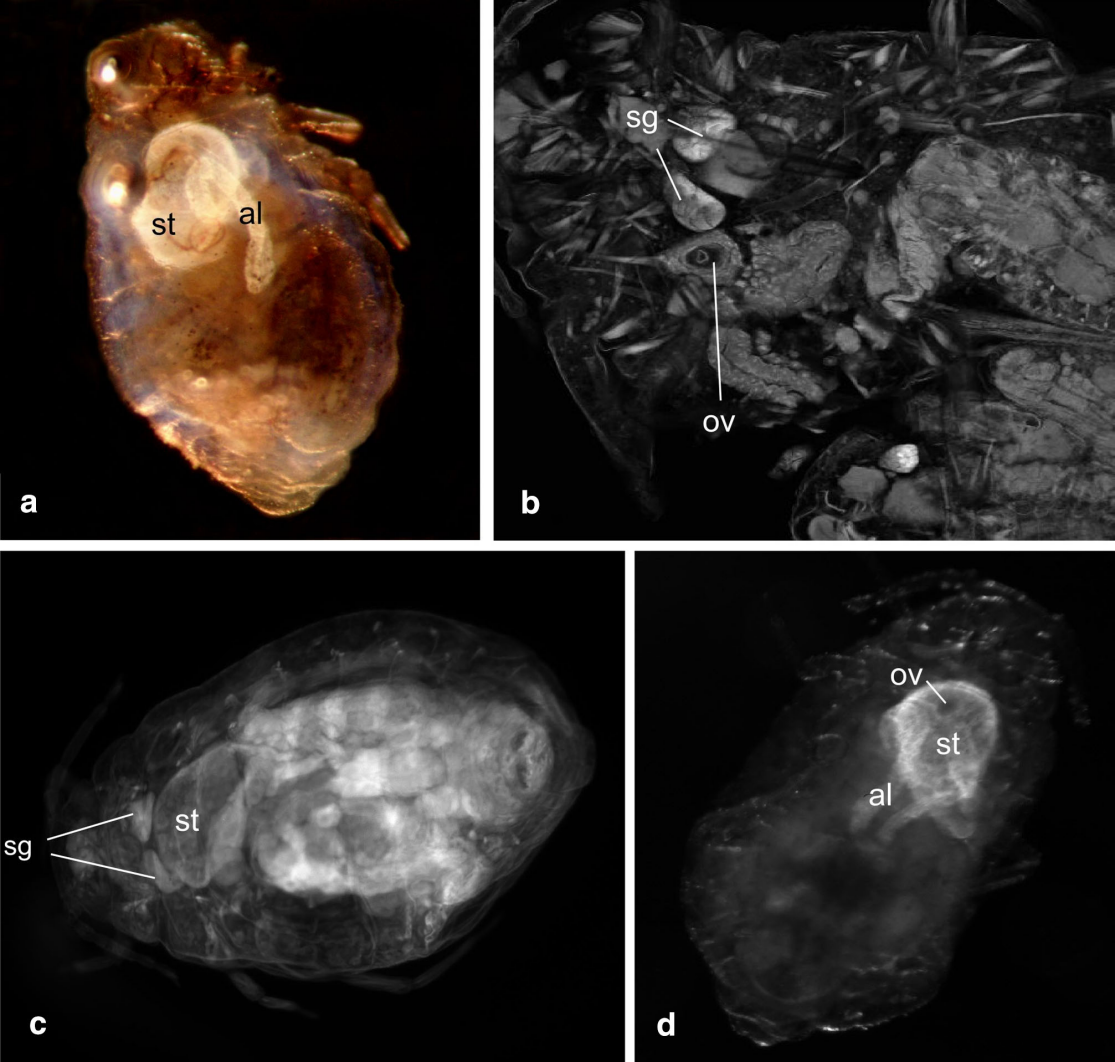
Alimentary tract of *G*. *utricularia*: **a** cleared specimen in oblique light microscopy; **b** view in CLSM, rhodamine-B stained; **c**, **d** cleared specimens in fluorescent microscopy, rhodamine-B stained; *sg* salivary glands, *ov* oesophageal valve, *st* stomach, *al* abdominal loop

**Fig. 5:**
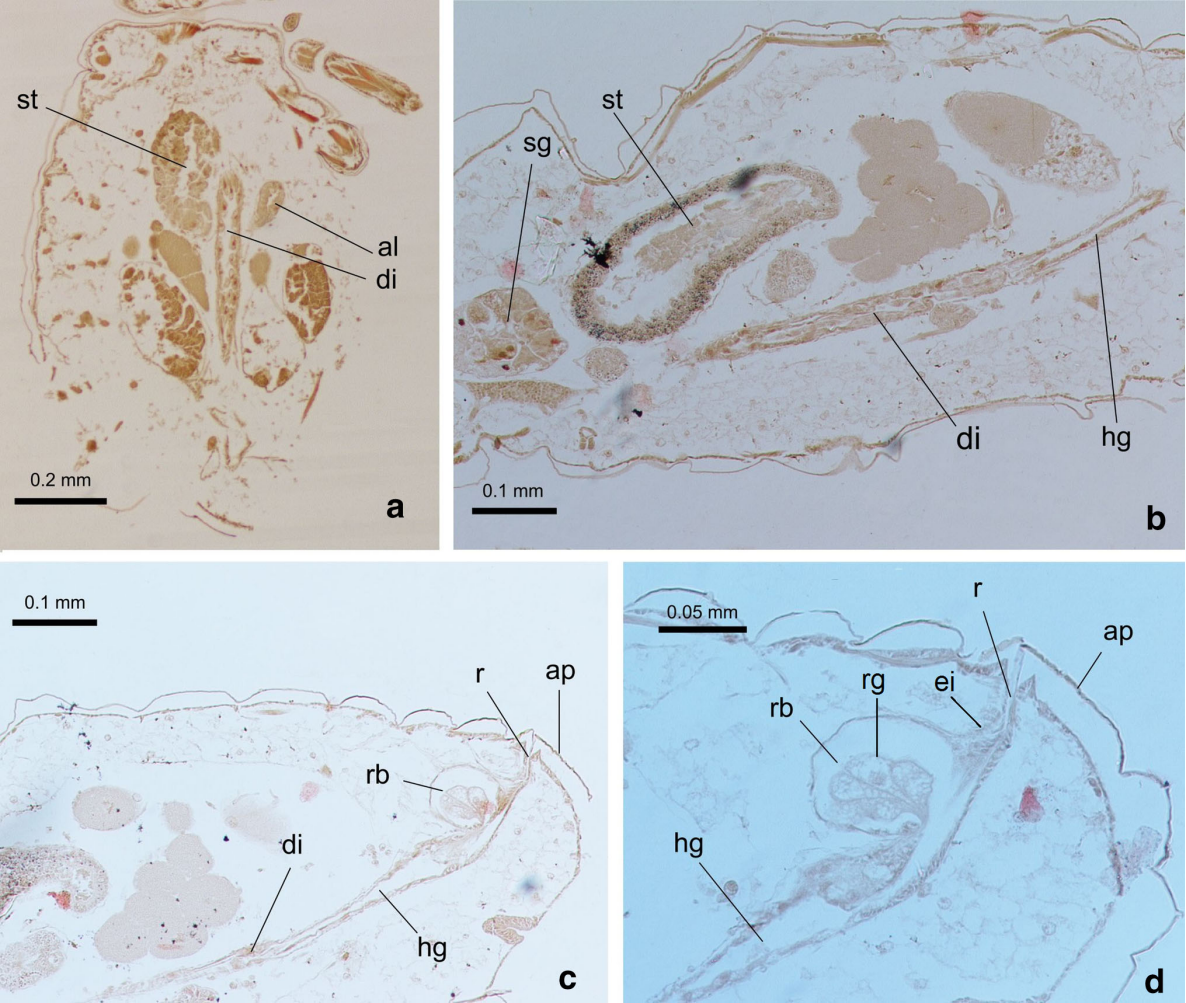
Alimentary tract of *G*. *utricularia*: **a** frontal section through the body; **b** longitudinal section through thorax and abdomen; **c**, **d** longitudinal section through posterior part of the abdomen; *sg* salivary gland, *st* stomach, *al* abdominal loop, *di* descending intestine, *hg* hindgut, *rb* rectal bladder, *rg* rectal gland, *ei* epidermal invagination, *r* rectum, *ap* anal plate

Genus *Geoica* Hart, 1894 is an Old World genus, also from the subfamily Eriosomatinae, comprising about 11 species, most of them being host alternating, with primary host being *Pistacia* and secondary host being the roots of various grasses. Their life cycle is two years, and on their primary host, they live in galls (Heie 1980; Blackman and Eastop 2006). In Central Europe, the anholocyclic population occurs, with parthenogenetic way of reproduction throughout the year. There are, however, data concerning *Geoicasetulosa* (Passerini, 1860), reporting the absence of stomach and crenated intestine (Ponsen 1991, 2006). The digestive system of *G*. *setulosa* consists of a foregut, which is closed at its posterior end, and a blindly starting descending intestine (Fig. 1). It seemed to be odd, taking into account the necessity for constant resorption of vast quantity of protein-poor food (Cristofoletti et al. 2003). Moreover, *Geoica* spp. are obligatorily mutualistic species on their secondary hosts and require the presence of ants, for which they produce honeydew (Heie 1980; Depa and Wojciechowki 2008). These species have even the anal plate developed into a trophobiotic organ (modified anal plate), enabling proper positioning of the honeydew droplet (Figs. 2, 3, 4, 5).

The aim of the study was to examine the structure of the alimentary tract of closely related species—*G. utricularia*, whose rectal bladder was studied by Ponsen (1991), to verify data on the reported lack of the stomach and crenated intestine. *G*. *utricularia* was used because it is more common and abundant, and both species differ only slightly in their morphology and biology. In *G*. *utricularia,* the anal plate is elongated, almost rectangular, with many setae dispersed on its entire surface, contrary to *G*. *setulosa*, where there are two rows of long setae. The purpose of these setae (in both species) is to keep the droplet of honeydew until it is collected by worker ant. Their host plants, life mode and relations with ants are in their anholocyclic range very similar and significantly overlap.

## Materials and methods

### Material

The material was collected in October and November 2014 on moist meadows in the vicinity of Piekary Śląskie, in southern Poland. In total, 32 individuals, apterous viviparous females of anholocyclic population on secondary host of *G*. *utricularia,* were collected, identified with the Heie’s key (1980).

### Methods

#### Histological preparations

In order to analyse the anatomical structure of the digestive system of *G*. *utricularia*, the paraffin method was applied. The material was collected into Eppendorf microtubes containing 70 % ethanol for keeping and preserving collected specimens. Next, insects were dehydrated in increasing concentration of ethanol (90, 96, 100 %). In order to ensure transparency, specimens were kept in methyl benzoate for one night. Then, material was consecutively transferred to benzene, benzene with paraffin (in proportions 2:3 and 1:2), paraffin I (melting point: 56 °C) and finally to paraffin II (melting point: 60 °C), where it was kept over night. After this process, material was immersed in paraffin II. The bars obtained were sectioned into 5 µm strips, which were stuck on slides in a 0.5 % gelatine solution at temperature 50–52 °C. Then, the slides were dried in 37 °C.

Slides were next deparaffined in xylene and treated with a series of ethanol solutions (100–60 %). They were rinsed in distilled water, stained with Ehrlich’s acid hematoxylin for about 20 min, rinsed again and differentiated with xylidine ponceau. After this process, preparations were treated with series of ethanol solutions (60–100 %), rinsed twice in xylene and embedded in Canadian balm or DPX.

This process was applied to 15 individuals of *G*. *urticularia*. Histological preparations were prepared: cross-sections from six individuals and longitudinal sections from nine individuals. In total, 49 microscopic slides were made, including 26 preparations of cross-section and 23 of longitudinal section. The cross-section series were also used to reconstruct the course of alimentary duct and in some cases the number and shape of cells.

#### Mounting of the whole tract

The specimens of *G*. *utricularia* were put into a droplet of 30 % ethanol, on the microscopic glass, and with the mounting needles, the whole alimentary tract was extracted from the body. The extracted organs were preserved in glycerol and mounted, to make all the anatomical structures visible at the stereomicroscope. A total number of 13 preparations were made using this technique.

The documentation was prepared using Nikon Eclipse E6000, with measurements made by Lucia net program. The pictures of translucent specimens were taken under stereomicroscope equipped with monochromatic camera Axio Cam programmed with Axio Vision.

#### Visualisations

In order to investigate localization and structure of gastric tract in *G*. *utricularia,* specimens were examined with oblique light microscopy, fluorescence microscopy and confocal laser scanning microscopy (CLSM). Before the examination, collected specimens were fixated overnight in cold 4 % PFA (paraformaldehyde) and subsequently underwent dehydration in graduated ethyl alcohol series, then optical clearing in methyl salicylate.

For fluorescent and CLSM imaging, specimens were prior to dehydration treated with 30 % hydrogen peroxide for 24 h and thereafter stained with Rhodamine-B before clearing in methyl salicylate. The staining with Rhodamine-B was applied for visualizing elastic tissues in CLSM (Shelley 1969). All reagents used in procedures had analytical grade purity. Fluorescent and oblique light imaging was performed with Carl Zeiss SteREO Lumar.V12 stereomicroscope and CLSM with Olympus FV1000 confocal system. Acquired images were processed in FIJI open source image processing package (Smolla et al. 2014).

## Results

The alimentary tract of *G*. *utricularia* begins with the external mouthparts and runs through the body cavity without any break or separately located organs. It ends at the anal orifice located above the dorsal edge of the anal plate (Figs. 2a–c, 5c, d).

The foregut (Figs. 2a, 3a) is a thin straight tube, about 27 μm long, with the diameter of 0.01–0.02 μm. In the prothorax, it runs between the two main salivary glands (Fig. 3c), which are built of ca. 20 cells. They are in shape of fan, bottle or other and comprise one or two nuclei located in central or in basal position in the cell, which is typical of exocrine glands (Fig. 3a–c). Their diameter is 0.08–0.12 μm. The wall of the foregut is made up of one layer of small flat epithelial cells, without any folds into its lumen. In the thorax, the foregut joins the stomach, by invagination into the cavity of stomach (Figs. 3b, d; 4b, d).

The midgut is the longest part of the alimentary tract and consists of the stomach, crenated and descending intestine.

The stomach begins in the second segment of thorax (mesothorax) (Figs. 2c, 3d) and extends towards the abdomen, slightly dorsally within the body. In longitudinal section, it has a shape of ventro-dorsally flattened bag (Figs. 4c, 5b), constricting gradually along its length. In its widest part, it has a diameter of 0.24–0.30 μm, and its length varies from 0.43 to 0.49 μm. The stomach is made up of two types of cells. The anterior part of stomach is built up of elongated, cone-shaped or finger-shaped cells, adhering to each other with lateral membranes at the cell bases (Figs. 3a, b, 5a, b), with their nuclei also elongated (Fig. 3a, b). Their free tips, directed towards the cavity of stomach, broaden the internal surface of the stomach. The cytoplasm of these cells comprises visible granules, which altogether indicates their secretive function.

The cells of the remaining distal part the stomach (Fig. 3b) are flat and hexagonal in shape and possess oval nuclei. The stomach narrows and opens into the crenated intestine, which runs distally; then, it loops towards the head (Figs. 2a–c; 4a, d; 5a), adhering the ventral surface of the stomach, and again loops towards the end of abdomen and opens into the descending intestine.

The descending intestine is a straight tube with thin walls, running through abdomen, and its wall is made up of flattened epithelial cells throughout its length (b, c). At its distal end, the descending intestine is broadened, and a well-developed rectal bladder is formed (Fig. 5c, d). It is positioned dorsally and has a roundish bubble shape, with a few secretive cells in its cavity. These cells are fan-shaped and secrete into the bladder (Fig. 5d). The rectal bladder opens into the epidermal invagination. It is a straight tube opening with the anal orifice.

## Discussion

The study showed that the alimentary tract of *G*. *utricularia* has the typical organization for this subfamily of aphids (Eriosomatinae), and the existing description of this system in *G*. *setulosa* is incomplete (Ponsen 1991, 2006). The organization of *G*. *utricularia* is quite similar to that of *Fordaformicaria* (von Heyden 1837), another representative of Erisomatinae. The presence of doubled nuclei in some of the cells in salivary glands is not surprising, since it was proved to occur also in other aphid families (Ponsen 2015), except of Eriosomatinae, which were not studied in this respect. The existing differences concern mainly the lengths of particular segments of the tract. Due to the lack of filter chamber, the adhering of the loops of crenated intestine to the ventral wall of the stomach may play the role of osmoregulation, as hypothesized for the pea aphid (Rhodes et al. 1997).

Another significant difference with other species of Eriosomatinae is the shape and position of rectal bladder. In *G*. *utricualria,* it is positioned dorsally, with a few cells encapsuled within the bladder, similarly to closely related *Smynthurodesbetae* Westwood, 1849 (Ponsen 1991). In *F*. *formicaria* and *F*. *marginata* (Koch 1857) (Ponsen 2006), the rectal bladder consists of a ring of cells of polygonal shape, surrounding the hindgut near the rectum. It concerns only females of *Forda* spp., because the presence of rectal bladder was not confirmed in males of this genus.

The role of rectal bladder is not sufficiently recognized. It is probably homologous with Malphigian tubules (Ponsen 1991), which have disappeared during the evolution of aphids. Its possible role is that it takes part in regulation of the honeydew composition (Dixon 1998), which contains carbohydrates, especially disaccharides: raffinose and melecitose, synthesized in aphid intestine, and their presence may play role in mutualistic relation of aphids and ants. In particular, the most attractive of ants is the honeydew rich in melecitose (Fisher and Shingleton 2001). Aphids producing honeydew with 30–70 % of melecitose (e.g. *Metopeurumfuscoviridae* Stroyan 1950) are more often ant attended than those not having melecitose in their honeydew (e.g. *Macrosiphumeuphorbiae* (Thomas 1878) or *Macrosiphoniellatanacetaria* (Kaltenbach 1843)) (Völkl et al. 1999; Fischer and Shingleton 2001). Similarly in a single genus, *Chaitphoruspopuleti* (Panzer 1804), with honeydew rich in melecitose, is more often attended by ants than *C*. *tremulae* (Koch 1854), with lower amount of melecitose in its honeydew. Taking into account the high level of myrmecophily of both *Forda* spp. and *Geoica* spp., the presence of well-developed rectal bladder may serve the purpose of regulating the composition of honeydew.

As a conclusion, it must be stated that the alimentary tract of apterous viviparous female of anholocyclic population of *G*. *utricualria* consists of all anatomical organs typical of aphid subfamily Eriosomatinae and constitutes a continuous tube. It is unlikely that *G*. *setulosa* may have differently built alimentary tract, since existing data on its foregut and hindgut fully agree with that of presented study on *G*. *utricularia*. However, taking into account complicated life cycle and heteroecy of *Geoica* spp,. there is essential need for further studies to confirm whether the situation of *Geoica* spp. (and also various morphs of species in this genus) is similar to that of *G*. *utricularia* or to that described by Ponsen.
